# Contribution of Pore-Connectivity to Permeation Performance of Silicalite-1 Membrane; Part II, Diffusivity of C_6_ Hydrocarbon in Micropore

**DOI:** 10.3390/membranes11060399

**Published:** 2021-05-27

**Authors:** Motomu Sakai, Yukichi Sasaki, Takuya Kaneko, Masahiko Matsukata

**Affiliations:** 1 Research Organization for Nano & Life Innovation, Waseda University, 513 Wasedatsurumaki-cho, Shinjuku-ku, Tokyo 162-0041, Japan; mmatsu@waseda.jp; 2 Nanostructures Research Laboratory, Japan Fine Ceramics Center, 2-4-1 Atsuta-ku, Nagoya-shi, Aichi 456-8587, Japan; sasaki@jfcc.or.jp; 3 Department of Applied Chemistry, Waseda University, 513 Wasedatsurumaki-cho, Shinjuku-ku, Tokyo 162-0041, Japan; t.kaneko.waseda@gmail.com; 4 Advanced Research Institute for Science and Engineering, Waseda University, 513 Wasedatsurumaki-cho, Shinjuku-ku, Tokyo 162-0041, Japan

**Keywords:** silicalite-1, membrane, separation, diffusion, adsorption, micropore, connectivity

## Abstract

This study investigated the permeation behaviors of *n*-hexane and 2-methylpentane through two-types of silicalite-1 membranes that have different pore-connectivity. The permeation mechanisms of these hydrocarbons were able to be explained by the adsorption–diffusion model. In addition, the fluxes through silicalite-1 membranes could be expressed by the modified Fick’s first law. The hydrocarbon fluxes through S-1_S_ with better pore-connectivity were ca. 3–20 times larger than those through S-1_M_ with poor pore-connectivity. For these membranes with different pore-connectivity, the activation energy of diffusion of *n*-hexane was 17.5 kJ mol^−1^ for the membrane with better pore-connectivity and 18.0 kJ mol^−1^ for the membrane with poorer pore-connectivity, whereas for 2-methylpentane it was 17.9 and 33.0 kJ mol^−1^, respectively. We concluded that the pore-connectivity in silicalite-1 membrane significantly influences the molecular diffusivities.

## 1. Introduction

Zeolite is a promising membrane material for hydrocarbon separations owing to its unique adsorption and molecular-sieving property. MFI-type zeolite membranes exhibit superior separation performances against hydrocarbon mixtures, such as butane isomer [1], hexane isomer [2] and xylene isomer [3,4,5]. Improvement of the permeation property is a big challenge for the development of the zeolite membrane, because hydrocarbon fluxes through zeolite membranes are too still low for practical use.

It is well-known that the permeation performance of the zeolite membrane is strongly affected by the membrane structure, such as the thickness and orientation of crystals. Previously, some strategies for flux improvement have been invented as introduced in part I. Attempts to control crystal orientations by seeding conditions and secondary growth conditions have often been made [6,7,8,9,10]. Further, unique methods were proposed to reduce membrane thickness [11,12].

We have focused our attention on grain boundaries that are formed by the collision of crystals in the course of the formation of the zeolite membrane. In part I, we prepared two types of silicalite-1 membranes and carefully investigated the microstructures of these membranes by using nano-permporometry, adsorption tests and permeation tests [13]. As a result, we concluded that the micropore volumes and effective pore size were strongly influenced by the existence of grain boundaries across the direction of molecular permeation. In addition, we defined pore-connectivity based on the results of adsorption tests. The pore-connectivity is the percentage of the micropores that are not narrowed and/or blocked, but that maintain their original pore size.

The study of adsorption and diffusion in a zeolite membrane is very important to understand the permeation and separation properties. The permeation flux of a molecule through a zeolite membrane was generally determined by the adsorption and the diffusion properties in the membrane [14,15,16]. Bakker et al. reported that gas permeation through their silicalite-1 membrane can be described with the enthalpy of adsorption and the activation energy for diffusion [14]. Nomura et al. also explained the transport mechanism of ethanol/water mixture through their silicalite-1 membrane with the adsorption–diffusion model [15]. While the adsorption properties of hydrocarbons in our silicalite-1 membranes were evaluated in part I, the effect of pore-connectivity on the diffusivity of a molecule is still an open question.

In this study, the permeation properties of C_6_ hydrocarbons were evaluated circumstantially in wide ranges of membrane temperatures and partial pressures. The two types of silicalite-1 membranes that have pore-connectivity of 60 and 47% were used for comparison [13]. The permeation properties were analyzed based on the adsorption–diffusion model, in order to discuss the effect of pore-connectivity on the diffusivity of hydrocarbons in the silicalite-1 membranes.

## 2. Materials and Methods

### 2.1. Membrane Preparation

Two types of silicalite-1 membranes (membrane S-1_S_ and S-1_M_) were prepared on an outer surface of a tubular α-alumina support (i.d. = 7.0 mm, o.d. = 10 mm, length = 30 mm, average pore size = 150 nm, NORITAKE Ltd., Nagoya, Japan) by a secondary growth method.

The synthesis solution of 10SiO_2_:1.6TPAOH:440H_2_O:40EtOH:0.04Na_2_O was hydrothermally treated at 373 K for 24 h to obtain the seed crystals for S-1_S_. Seed crystals were supported on a tubular support by a dip coating method. A seeded support was immersed in synthesis solution with the molar composition of 10SiO_2_:1.2TPAOH:660H_2_O:80EtOH, and then hydrothermal treatment was carried out at 373 K for 7 days to form a membrane, S-1_S_. S-1_M_ was prepared in essentially the same manner as S-1_S_. The molar composition of the synthesis solution and the conditions of hydrothermal treatment were modified. More detailed descriptions of synthesis procedures of S-1_S_ and S-1_M_ can be found in part I.

S-1_S_ and S-1_M_ are silicalite-1 membranes with a single-layer crystal and a multi-layer crystal, respectively. Wedge-shaped crystals were present on the support surface with a thickness of ca. 4.0 μm in membrane S-1_S_. In contrast, membrane S-1_M_ was composed of a number of small crystals with a thickness of ca. 2.5 μm. As described in part I, the micropore volume and effective pore size in S-1_S_ are greater than those in S-1_M_. In addition, pore-connectivity through S-1_S_, 60%, is better that that through S-1_M_, 47%. The typical FE-SEM images of the two types of silicalite-1 membranes were also shown in part I.

### 2.2. Vapor Permeation Test

To investigate the diffusivity of hydrocarbon in micropores through two types of silicalite-1 membranes, permeation tests were carried out in wide temperature and partial pressure ranges. We can evaluate an effect of molecular size on diffusivity by using hexane isomers, *n*-hexane (ca. 0.43 nm [17]) and 2-methylpentane (ca. 0.50 nm [18]), as feed materials.

**Figure 1:**
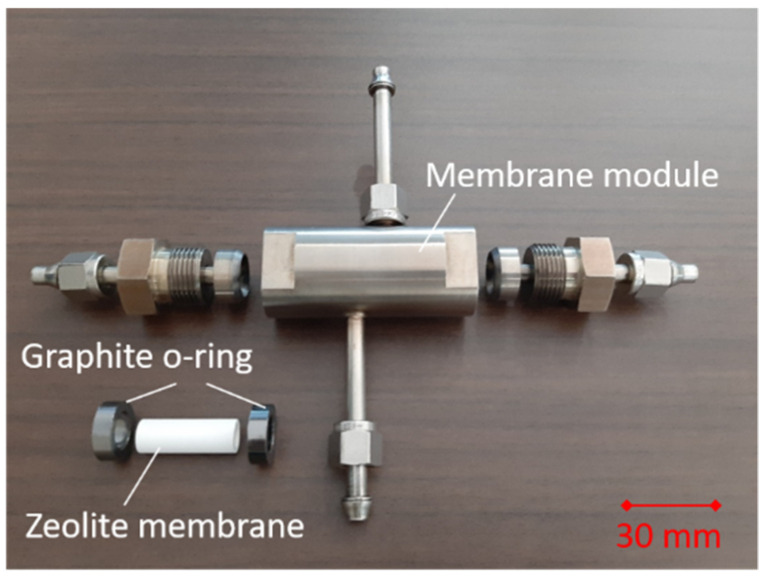
The photo of the membrane module for the vapor permeation test.

Permeation tests took place using a home-made apparatus in a vapor permeation mode. Figure 1 shows the photo of the membrane module for the vapor permeation test. A silicalite-1 membrane was fixed with a graphite o-ring into the membrane module. Either *n*-hexane or 2-methylpentane were pumped into a preheater to vaporize and then fed to the outer surface of a tubular membrane. The partial pressure of hydrocarbon vapor was adjusted by controlling the flow rate of the dilution gas, helium. The permeate side was swept with flowing helium. Both the feed and permeate sides were kept at atmospheric pressure, meaning that only permeation due to concentration gradient was evaluated.

To exclude an effect of concentration polarization on the closest surface of the membrane, sufficiently large amount of feed and sweep gas were flowed. Specifically, the ratio of permeation flow rate to feed flow rate was kept less than 5% and the partial pressure of hydrocarbon in the permeate side was diluted to less than 0.5 kPa in all conditions.

The flow rate was determined by a gas chromatograph (GC-8A, Shimadzu Corp., Kyoto, Japan) using the internal standard gas, methane. Permeation flux *J* was calculated using the following Equation (1);

*J* = *uA*^−1^ (1)

where *u* is the permeation flow rate (mol s^−1^) and *A* is the effective membrane area (m^2^), 6.28 × 10^−4^ m^2^.

## 3. Results and Discussion

### 3.1. Vapor Permeation Properties of Silicalite-1 Membrane for C_6_ Hydrocarbons

*n*-Hexane and 2-methylpentane fluxes through two types of silicalite-1 membranes in unary systems were evaluated to investigate the effect of pore-connectivity on the permeation property. The partial pressure of hydrocarbon vapor was controlled from 10 to 50 kPa in the temperature range of 373 to 653 K.

**Figure 2:**
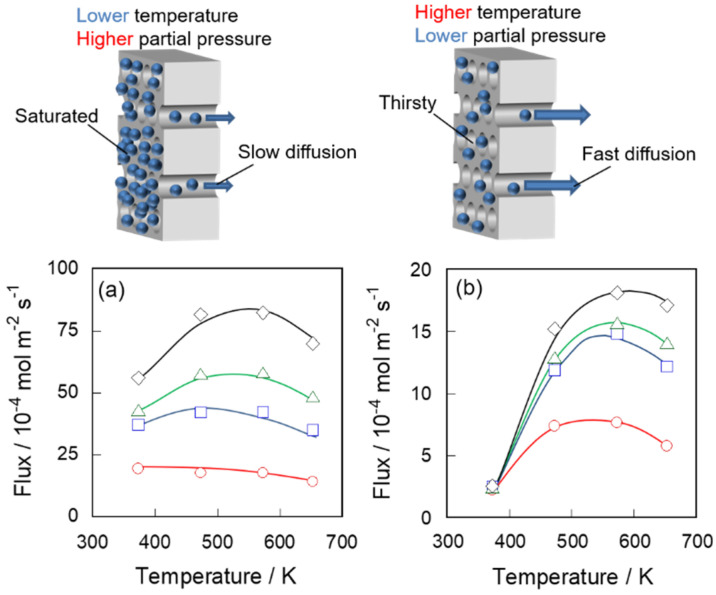
Membrane temperature dependency of *n*-hexane fluxes through (**a**) S-1_S_ and (**b**) S-1_M_. Partial pressures of *n*-hexane are at ○, 10; □, 25; ▵, 35 and ◊, 51 kPa.

Figure 2 shows the permeation fluxes of *n*-hexane through the silicalite-1 membranes. The fluxes through two types of membranes increased with increasing partial pressure under all conditions. The fluxes had maximum values at given a temperature. In addition, the temperature at which the *n*-hexane fluxes had maxima became higher at higher partial pressure conditions. These permeation behaviors were very similar between S-1_S_ and S-1_M_, although the permeation fluxes through S-1_S_ were 3–20 times larger than those through S-1_M_. For instance, the *n*-hexane fluxes at 50 kPa through membranes S-1_S_ and S-1_M_ at 573 K were 8.83 × 10^−3^ and 1.81 × 10^−3^ mol m^−2^ s^−1^, respectively.

The behaviors of *n*-hexane permeation can be explained by the typical surface diffusion model and quantitatively analyzed by the adsorption–diffusion model described in the introduction [14]. At a lower temperature, an adsorbed phase is present in the zeolite pore. In this adsorbed phase the mass transport takes place by molecules moving between adsorption sites. In such a surface diffusion model, the *n*-hexane fluxes are determined almost entirely as a product of the adsorption amount on and the diffusion rate in the membrane.

The partial pressure affects the adsorption amount; the adsorption amount was increased naturally by increasing partial pressure. In contrast, the membrane temperature had both negative and positive effects on the adsorption amount and the diffusivity, respectively. Therefore, the flux had maxima at the appropriate temperature, at which the adsorption amount and the diffusivity balanced. At higher partial pressures, the temperature showing maximum flux rose higher because the adsorption amount remained relative at a higher temperature. These permeation behaviors are the rule rather than the exception, as mentioned in the introduction [14,15,16].

**Figure 3:**
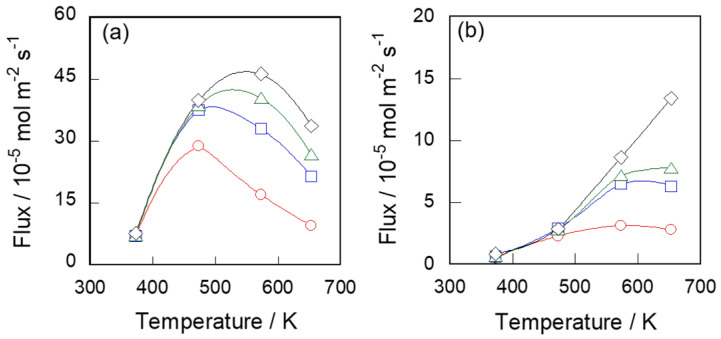
Membrane temperature dependency of 2-methylpentane fluxes through (**a**) S-1_S_ and (**b**) S-1_M_. Partial pressures of 2-methylpentane are at ○, 10; □, 25; ▵, 35 and ◊, 51 kPa.

Figure 3 shows the permeation properties of these membranes for 2-methylpentane. 2-Methylpentane fluxes were a magnitude lower than those of *n*-hexane owing to the large molecular size of ca. 0.50 nm [18] compared with *n*-hexane, ca. 0.43 nm [17]. The fluxes through S-1_S_ and S-1_M_ at 573 K at 50 kPa were 4.63 × 10^−4^ and 8.63 × 10^−5^ mol m^−2^ s^−1^, respectively. The temperature and partial pressure dependency of 2-methylpentane fluxes were similar to those of *n*-hexane.

The difference in pore-connectivity between S-1_S_ and S-1_M_ was already discussed in part I. From the results of the nano-permporometry test and the adsorption test in part I, we concluded that the narrowing and obstruction of micropores occurred along grain boundaries. The poor pore-connectivity of S-1_M_ compared with S-1_S_ would lead to the small permeation fluxes of *n*-hexane and 2-methylpentane.

### 3.2. Modeling of Hydrocarbon Permeation through Silicalite-1 Membrane

The results of the permeation tests suggested that hydrocarbon fluxes were determined by the adsorption amount and the diffusion rate. Next, we represented the fluxes using an equation that includes the terms of adsorption and diffusion, for quantitative evaluation of the effects of these factors on permeation flux.

It was defined that both silicalite-1 membranes, S-1_S_ and S-1_M_, have few defects, as described in part I. Hence, we assume that all molecules permeate only through micropores.

Fick’s first law, the following equation, is used as a basic idea in this study.

*J* = −*D*·*dC*/*dx* (2)

Additionally, we assume that the two factors that are the driving force of permeation are the difference in the adsorption amount between the feed and permeation side, and the concentration gradient in the membrane being linear. Then, Fick’s first law forms the following Equation (3):

*J* = −*D* (*V_F_* − *V_P_*)/*dx* (3)

*V_P_*, the adsorption amount on the permeation side, is approximated by zero because the permeate side was swept with the amount of inert gas in the permeation test.

The adsorption of hydrocarbons on zeolite follows the Langmuir model, as described in part I. Therefore, Equation (4) represents *V_F_*, adsorption amount on the feed side:

*V_F_* = *abP*·(1 + *bP*)^−1^ (4)

where *a* and *b* are the saturated adsorption amount (mol m^−3^) and the adsorption equilibrium constant (-), respectively. Then Equation (5) was derived from Equations (3) and (4) by using *D*′ (= *aDL*^−1^).

*PJ*^−1^ = *D*′^−1^*P* + *b*^−1^*D*′^−1^ (5)

*D*′ is the term including the diffusion coefficient. If fluxes are able to be represented by Equation (3), plots as *P J*^−1^ against *P* should be linear. In addition, *D*, which means the diffusion coefficient in the membrane, can be determined from the inverse of the slope of the line, saturated adsorption amount and membrane thickness.

**Figure 4:**
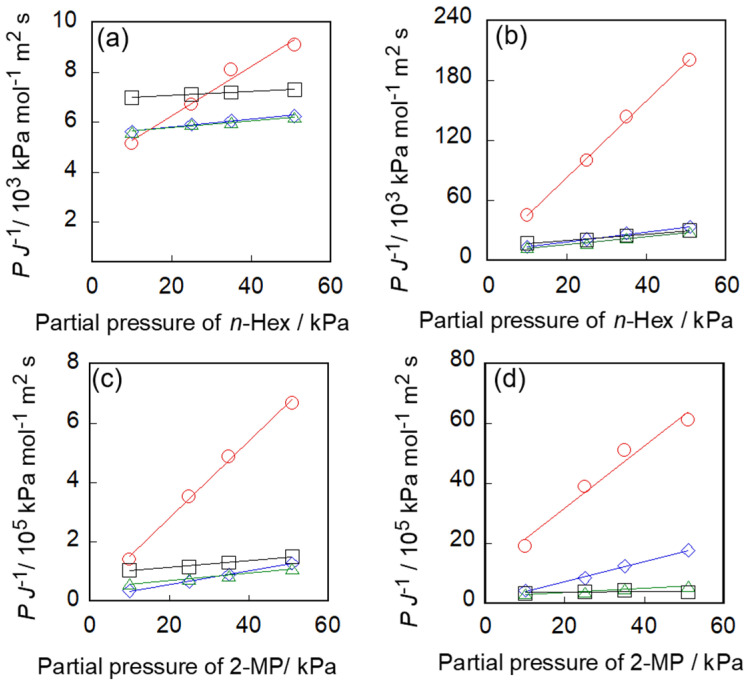
The plots of *PJ*^−1^ against *P*. (**a**,**b**) *n*-hexane through S-1_S_ and S-1_M_. (**c**,**d**) 2-methylpentane through S-1_S_ and S-1_M_. ○, 373; □, 473; ▵, 573 and ◊, 653 K.

Figure 4 shows *P J*^−1^ against *P* by using the values in Figure 2 and Figure 3. The plots under the same temperature lie on a straight line, indicating that hydrocarbon fluxes through the silicalite-1 membrane in unary systems can be represented by Equation (3). As a result, the permeation behaviors of hydrocarbons through two types of silicalite-1 membranes can be exhibited quantitatively by the adsorption–diffusion model. In addition, the driving force of permeation could be explained by the difference of the adsorption amount between feed and permeate sides, as shown in the modified Fick’s first law, Equation (3).

### 3.3. Diffusivities in Two Types of Silicalite-1 Membranes

*D*′, the term including diffusion coefficient, was defined as the inverse of the slope of lines in Figure 4, mentioned above. Here, we calculated the diffusion coefficient, *D*, by multiplying *D*′ by membrane thickness, *L*, and dividing by the saturated adsorption amount, *a*. The thicknesses of the membranes S-1_S_ and S-1_M_ were estimated by observation of FE-SEM as 4.0 and 2.5 μm, respectively. The values of the saturated adsorption amounts shown in part I were used for the calculation. The amounts of *n*-hexane adsorbed in S-1_S_ and S-1_M_ were 1.73 and 1.35 kmol m^−3^ (=21.1 and 16.5 cm^3^ (STP) g^−1^), respectively. The adsorbed amounts of 2-methylpentane in S-1_S_ and S-1_M_ were 0.984 and 0.780 kmol m^−3^ (=12.0 and 9.52 cm^3^ (STP) g^−1^), respectively.

**Figure 5:**
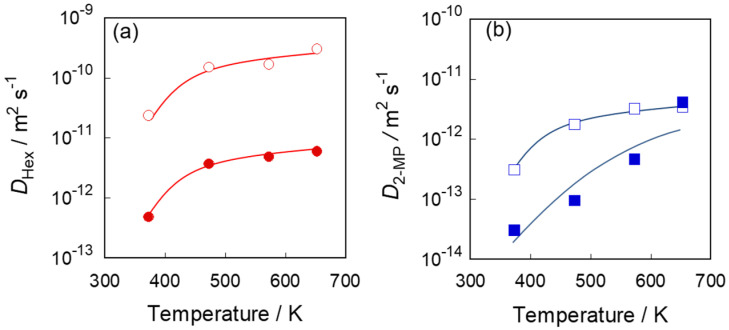
The temperature dependency of the diffusion coefficient of (**a**) *n*-hexane, (**b**) 2-methylpentane. Open and solid symbols show S-1_S_ and S-1_M_, respectively.

Figure 5 shows the calculated diffusion coefficients of *n*-hexane and 2-methylpentane through S-1_S_ and S-1_M_. The diffusion coefficient of *n*-hexane through S-1_S_ was two orders of magnitude larger than that through S-1_M_. For example, the diffusion coefficients through S-1_S_ and S-1_M_ at 373 K were 2.3 and 0.048 × 10^−11^ m^2^ s^−1^. The diffusion coefficients of *n*-hexane through both membranes were strongly affected by the membrane temperature. The diffusion rates at 473 K and 653 K were ca. 7-times and ca. 12-times larger than that at 373 K. In addition, the diffusion coefficient of *n*-hexane was an order of magnitude larger than that of 2-methylpentane. The difference in diffusivity between *n*-hexane and 2-methylpentane could be caused by the difference in molecular size.

Table 1 lists the self-diffusivities of *n*-alkanes in powdery crystals of MFI-type zeolite to compare with the values determined in this study [19,20,21,22,23,24]. The self-diffusivities in previous reports were from 0.31 to 45 × 10^−11^ m^2^ s^−1^ ca. 300 K. Values of diffusivities have large differences among measurement methods. The diffusivity through S-1_S_ defined from the permeation test in this study was relatively close to literature values.

**Figure 6:**
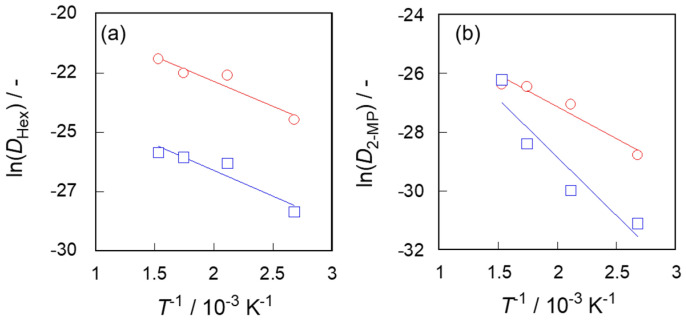
Arrhenius plot for diffusion of (**a**) *n*-hexane and (**b**) 2-methylpentane. ○, S-1_S_; □, S-1_M_.

Finally, the activation energy for diffusion penetrated through silicalite-1 membranes was evaluated. Figure 6 shows the Arrhenius plots for calculated *D* values. The activation energy of the diffusion of n-hexane was 17.5 kJ mol^−1^ for the membrane with better pore-connectivity and 18.0 kJ mol^−1^ for the membrane with poorer pore-connectivity, whereas for 2-methylpentane it was 17.9 and 33.0 kJ mol^−1^, respectively.

The diffusion coefficients and the activation energy of diffusion elucidated that S-1_M_ had greater diffusion resistance. It is strongly suggested that the narrowing and obstruction of micropores became a barrier of diffusion possibly because the micropore mismatch occurs at the crystal interfaces formed by the collision of growing crystals during the membrane formation process, as described in part I. Large molecules, whose size is close to the pore size, tended to be strongly affected by such pore-connectivity. Here we proposed that the preparing of membranes with good pore-connectivity, by the reducing of grain boundary, is an effective strategy to obtain high-permeable zeolite membranes.

## 4. Conclusions

Two types of silicalite-1 membrane with pore-connectivity values of 60 and 47% were prepared and their permeation performances for hydrocarbon were carefully evaluated. The permeation behaviors of *n*-hexane and 2-methylpentane could be simply explained by the adsorption–diffusion model. The diffusion coefficient through a membrane with better pore-connectivity was ca. 50-times larger than that through a membrane with poor pore-connectivity. The activation energy of diffusion in micropores was also significantly different between these two membranes; the activation energies of 2-methylpentane were 17.9 and 33.0 kJ mol^−1^ through the membranes. We suggest that preparing membranes with good pore-connectivity by reducing the grain boundary is an effective strategy to obtain highly permeable zeolite membranes.

## Figures and Tables

**Table 1:** Self-diffusivities of *n*-alkanes in MFI-type zeolite.

Methods	Temperature/K	Species	**Self-Diffusivity** /10^−10^ m^2^ s^−1^	References
MD	300	*n*-hexane	30	[18]
	300	*n*-butane	45	
MD	300	*n*-hexane	22	[19]
	300	*n*-butane	32	
Wicke–Kallenbach	303	*n*-butane	0.31	[20]
Pulse–response	-	*n*-butane	45.6	[21]
PFG NMR	- -	*n*-hexane *n*-pentane	2.9	[22]
			10	
Frequency–response	303 303	*n*-butane *n*-hexane	5.2 4.1	[23]
Unary permeation test	373 373	*n*-hexane	0.23	S-1_S_ (This work) S-1_M_ (This work)
		*n*-hexane	0.0048	

## Nomenclature

*a*	Saturated adsorption amount (mol m^−3^)
*A*	Effective membrane area (m^2^)
*b*	Adsorption equilibrium constant (-)
*C*	Concentration (mol m^−3^)
*D*	Diffusion coefficient (m^2^ s^−1^)
*D*′	Term including diffusion coefficient (=*aDL*^−1^)
*J*	Permeation flux (mol m^−2^ s^−1^)
*L*	Membrane thickness (m)
*P*	Partial pressure of hydrocarbon in feed side (Pa)
*R*	Gas constant (J mol^−1^ K^−1^)
*u*	Flow rate (mol s^−1^)
*V_F_*	Adsorption amount on the membrane in feed side (mol m^−3^)
*V_P_*	Adsorption amount on the membrane in permeate side (mol m^−3^)
